# Uncertainty Analysis and Metrological Validation of Raman Distributed Temperature Measurements in a Full-Scale Test Facility

**DOI:** 10.3390/s26092830

**Published:** 2026-05-01

**Authors:** Maxime Houvin, Rafik Moulouel, Pascal Borel, Didier Boldo

**Affiliations:** EDF R&D Prisme, 6 quai Wattier, 78400 Chatou, France; maxime.houvin@edf.fr (M.H.); pascal.borel@edf.fr (P.B.); didier.boldo@edf.fr (D.B.)

**Keywords:** GUM, uncertainty, temperature measurement, Raman distributed temperature sensor

## Abstract

**Highlights:**

**What are the main findings?**

- Uncertainty characterization of Raman DTS using a GUM-based approach.
- Application to a full-scale test facility.

**What are the implications of the main findings?**

- Provides a reliable framework to ensure traceable DTS temperature measurements.
- Helps improve accuracy in thermal field reconstruction in real experimental environments.

**Abstract:**

Raman Distributed Temperature Sensing (DTS) provides spatially distributed temperature measurements along optical fibers and is increasingly used for monitoring large-scale infrastructures and experimental facilities, enabling three-dimensional reconstruction of temperature fields. However, such measurements involve specific implementation constraints and may be affected by significant errors, with uncertainties influenced by factors such as calibration, environmental conditions, spatial resolution effects, and fiber positioning. Ensuring the metrological validity of Raman-based DTS measurements therefore requires a rigorous quantification of the associated measurement uncertainties. In this work, a complete uncertainty analysis of Raman-based DTS measurements is performed following the principles of the Guide to the Expression of Uncertainty in Measurement (GUM). A measurement model describing the relationship between Raman backscattered signals and temperature is established, and all relevant uncertainty sources are identified and quantified. The methodology is applied to a full-scale experimental facility equipped with a DTS interrogator and a dedicated calibration setup. Uncertainty propagation is performed using both first-order Taylor series expansion and Monte Carlo simulation, providing consistent results. The analysis shows that calibration uncertainty, spatial dispersion of the temperature field and fiber positioning within the reconstructed temperature field represent the dominant contributions to the combined uncertainty. The proposed approach provides a rigorous framework for the metrological qualification of Raman DTS systems and offers practical guidance for improving measurement reliability in distributed temperature monitoring applications.

## 1. Introduction

Distributed temperature sensing (DTS) based on Raman backscattering has become a widely used technique for spatially resolved temperature measurements over extended distances. It has been successfully applied in a broad range of domains, including environmental monitoring, hydrology, structural health monitoring, and industrial process control [1,2,3]. By enabling a large number of distributed measurements along a single optical fiber, Raman DTS allows temperature monitoring in both solid and gaseous media with high spatial coverage. This capability makes it possible to reconstruct temperature fields in complex environments and to obtain detailed three-dimensional representations of thermal distributions.

Unlike mapping using an array of point probes, such as thermocouples [4], which are difficult to implement and can significantly alter the measurement environment (notably via supports and connections), fiber optic temperature measurement techniques, which are generally small, insensitive to electromagnetic interference, resistant to harsh environments, and capable of distributed measurements, enable complete environmental mapping [5]. Although several methods exist, including Fiber Bragg Grating (FBG), Fabry–Perot Interferometric Sensors, and light scattering-based sensors [6], such as Raman, Brillouin, and Rayleigh scattering techniques, each with different performance characteristics and application domains [7], recent advances in distributed temperature sensors (DTS) using Raman interrogators, particularly in terms of improved accuracy and spatial resolution, have encouraged the use of this technology for temperature mapping using optical fibers [8,9,10].

Manufacturers of Raman interrogators provide uncertainty specifications and information on influencing factors ([11], for example) and various studies have been carried out on estimating the uncertainty of temperature measurements in the context of characterization benches [12,13]. However, in the context of a controlled measurement environment that is not primarily designed to characterize the uncertainty of the measurement chain, the identification and evaluation of uncertainty sources—particularly within a temperature sensor network—remain complex and require the structured implementation of a comprehensive uncertainty assessment methodology.

In this article, the uncertainty evaluation framework based on the Guide to the Expression of Uncertainty in Measurement (GUM) [14,15] is first introduced. The Raman distributed temperature sensing measurement model is then presented, followed by the identification and characterization of the main sources of uncertainty affecting Raman DTS measurements. Standard uncertainty estimators are proposed for the different components of the measurement chain, including calibration, spatial effects, temporal dispersion, and positioning uncertainties.

The methodology is finally applied to temperature measurements performed in the “Electrical Cabinet Room” test facility of the ZEPHYR laboratory, where distributed optical fibers are used to reconstruct temperature fields both in air and within concrete walls.

## 2. GUM Framework

**Figure 1:**
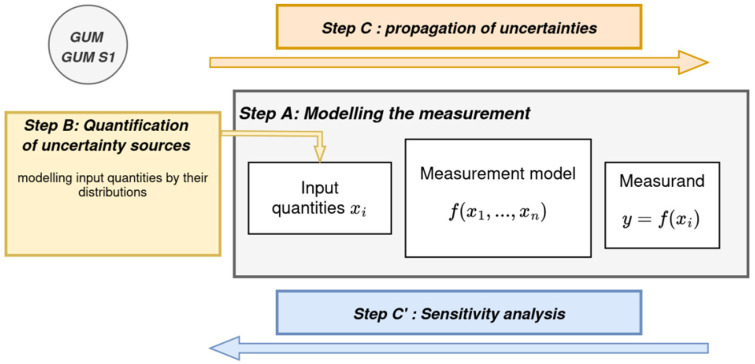
GUM-based framework for uncertainty evaluation in measurement.

The uncertainty analysis follows a structured methodology composed of three main steps, as shown in Figure 1:

- **Step A—Problem specification.**

This step consists of defining the measurement objective and the quantity of interest. A statistical or physical measurement model is established to describe the relationship between the input variables and the output quantity. The objective of the analysis is then defined, for example, the evaluation of the dispersion of the output variable or the probability of exceeding a given threshold.

- **Step B—Quantification of uncertainty sources.**

All input variables contributing to the uncertainty of the quantity of interest are identified and characterized by their statistical properties (standard deviations, probability distributions, etc.). These characteristics may be obtained either from experimental measurements (Type A evaluation) or from manufacturer specifications, prior studies, or expert judgment (Type B evaluation).

- **Step C—Propagation of uncertainties.**

The uncertainties associated with the input variables are propagated through the measurement model to estimate the uncertainty of the output variable. This can be performed using the law of propagation of uncertainty (first-order Taylor series approximation) or by Monte Carlo simulations, as recommended by the GUM and its Supplement 1.

- **Step C’—Sensitivity analysis.**

Finally, a sensitivity analysis is performed to rank the input quantities according to their contribution to the overall uncertainty and to identify the most influential sources.

## 3. Measurement Model

**Figure 2:**
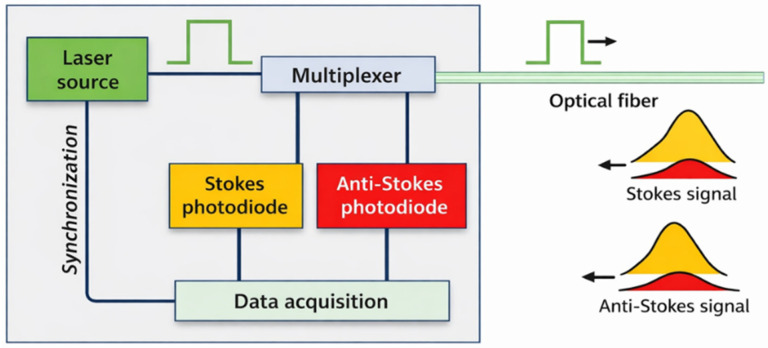
Simplified schematic of a Raman Distributed Temperature Sensing (DTS) measurement system.

Figure 2 illustrates the principle of DTS measurements. Raman interrogators send monochromatic laser pulses (of wavelengths 1064 or 1550 nm) and measure the backscattered power within the fiber, at two frequencies called the Stokes and the anti-Stokes peaks. The return time of the pulse is used to calculate the abscissa of the measured area along the fiber. At any instant t and at any point on the abscissa l, the power ratio between these two peaks is related to the temperature of the fiber by the following formula [11]:

(1) $$T\left(t,l\right)=T_{offset}+\frac{\gamma }{\ln\left(\frac{P_{s}\left(t,l\right)}{P_{as}\left(t,l\right)}\right)+C\left(t\right)-\Delta \alpha l}$$

where $P_{s}$ and $P_{as}$ are the measured intensities at the Stokes and anti-Stokes wavelengths, C is an intrinsic parameter of the instrument, and $T_{offset}$ and Δα are parameters to be calibrated by the user: $T_{offset}$ represents the constant measurement error that can be corrected by a reference measurement and Δα is the differential attenuation of the optical fiber at the wavelengths of $P_{s}$ and $P_{as}$.

**Figure 3:**
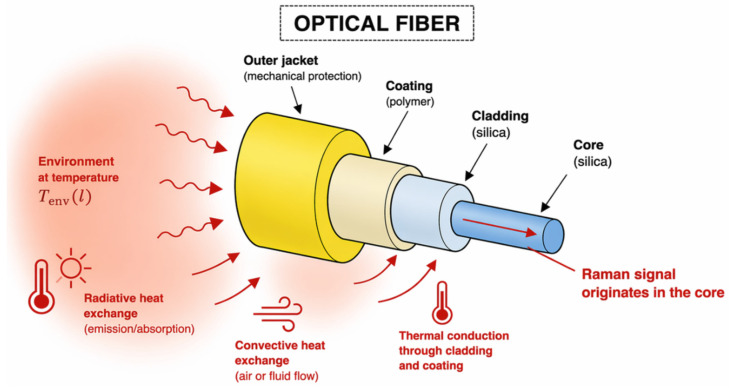
Main components of an optical fiber.

Figure 3 illustrates that the temperature-sensitive part of the optical fiber is associated with the silica core. All the elements constituting the optical fiber are positioned in an environment where they can be subjected to conductive (at the level of their protective layers and indirectly through contact with a material), radiative, or convective effects.

Through thermal conduction across the cladding and protective coating layers, the fiber reaches thermal equilibrium with its surrounding environment, as discussed in studies on heat transfer mechanisms in distributed temperature sensing systems [16]. As a result, the measured temperature reflects the local thermal state of the environment surrounding the fiber.

The main purpose of using this measurement technology is to construct temperature fields from distributed measurements, which allows several measurement points on the same fiber, depending on the interrogator’s sampling capacity. In this context, in addition to controlling errors induced by unwanted thermal effects, it is necessary to understand each local fiber measurement in terms of its spatial errors associated with the position of the measurement point in space and the influence of its close neighbors. Given the relatively long sampling times, this field measurement technology is better suited to averaged temperature measurements. The output quantity of interest is the time-averaged temperature at each optical fiber sampling point $T_{l}^{t}$ (temperature measured at linear position l along the optical fiber at time t).

Measurement requirements are expected in the form of a **two-sided 95% confidence interval around the target variable**.

While most DTS uncertainty analyses rely on the analytical temperature equation derived from the Raman scattering model, as in Des Tombe [17], the present study adopts a practical metrological perspective by addressing the complete measurement chain and propagating the dominant uncertainty sources at the temperature field level. The measurement model is expressed as follows:

(2) $$T_{measured}=T_{real}+\sum \varepsilon_{i}$$

where each $\varepsilon_{i}$ represents a contribution from the different sources of uncertainty affecting the measurement chain.

## 4. Identification and Quantification of Uncertainty Sources

The uncertainty associated with instantaneous temperature $T_{l}^{t}$ obtained by Raman Distributed Temperature Sensing (DTS) measurements originates from several elements of the measurement chain. The variable of interest depends on the performance of the interrogator, the efficiency and representativeness of the sensitive measurement parts (core of the optical fiber) with respect to the target measurement medium, and the spatial accuracy of the temperature fields evaluated.

From a metrological perspective, the main contributors can be grouped into four categories: uncertainties related to the interrogator, uncertainties related to the optical fiber, uncertainties associated with the interrogator–fiber interaction, and uncertainties introduced during the spatial reconstruction of the temperature field. The main sources of uncertainty identified in the DTS measurement chain are summarized in Table 1.

A comprehensive evaluation of the various sources of uncertainty is required to obtain a reliable final measurement uncertainty. When focusing on the interrogator–fiber subsystem as a partial measurement chain, although some contributors—such as the intrinsic characteristics of the interrogator, its environmental sensitivity (temperature, pressure, humidity, supply voltage, radiation), the performance of the acquisition electronics, or the accuracy of the internal reference used for temperature offset correction—are generally specified by the manufacturer, these specifications are insufficient when the device is used with a specific fiber and in a given thermal environment. In practice, temperature-dependent corrections and fiber-dependent parameters necessitate the calibration of the complete “interrogator + fiber” system. To characterize these contributions accurately, a dedicated calibration setup is required. This system should meet the following criteria:

- provide a thermally homogeneous environment equipped with at least one traceable reference temperature sensor;
- accommodate the entire test fiber or a representative segment of it;
- allow temperature variations over the full range expected during real measurements;
- enable the characterization of environmental variations affecting the interrogator itself.

In addition, regarding the intrinsic characteristics of the fiber itself, the uncertainty associated with the differential attenuation Δα of the fiber must be evaluated under realistic installation conditions, including bends, mechanical stresses, splices, and thermal gradients, all of which significantly influence attenuation. An alternative approach consists of using a double-ended configuration of the interrogator, in which the fiber is looped back to a second detection channel. By launching laser pulses alternately from each end of the fiber, this configuration enables a more accurate determination of the attenuation profile along the entire path.

Furthermore, the temperature measured in the fiber core may not be immediately representative of the surrounding medium. Imperfect thermal coupling, thermal transients, or mechanical constraints (e.g., when the fiber is embedded in a solid) can introduce additional bias. For this reason, an in situ validation is required to assess the thermal equilibrium between the fiber and the measurement environment and to quantify any residual measurement errors.

## 5. Experimental Case Study

### 5.1. Facility

**Figure 4:**
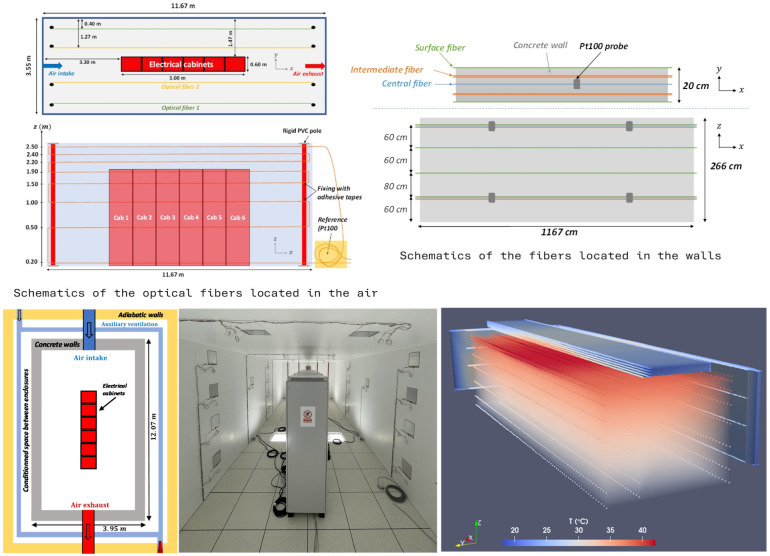
Picture and schematic of the Electrical Cabinet Room, along with the experimental reconstruction of temperature fields within the walls and the air of the testing facility.

To illustrate the proposed approach, we consider as a case study the measurement of temperature fields in both the walls and the air of the “Electrical Cabinet Room” test facility of the ZEPHYR laboratory. This experimental facility, described in detail in [18] and shown in Figure 4, consists of a test room built with concrete walls and covering a floor area of 40 m^2^. The room is equipped with a mechanical ventilation system and contains experimental electrical cabinets that are closed and naturally ventilated. These cabinets dissipate heat generated by several electric resistances distributed vertically along their height. The configuration is representative of an electrical and control-command room typically found on a nuclear site. To control the external boundary conditions applied to the envelope of the room (the concrete walls), the test chamber is enclosed within a second insulated structure. The interstitial space between the two enclosures is conditioned using an auxiliary ventilation system, ensuring a uniform air temperature in that space.

A Silixa Ultima-S interrogator, operating with a spatial sampling resolution of 12.6 cm, is used to measure temperature fields within both the walls and the air of the room. Approximately 1 km of optical fiber is embedded in the concrete walls and ceiling at various heights and depths. In addition, about 0.5 km of aluminum-coated optical fiber is deployed to measure air temperature within the room. The aluminum coating is used to reduce measurement bias induced by radiative heat transfer [5]. This configuration provides roughly 12,000 measurement points, arranged in horizontal segments at several elevations and symmetrically distributed along the x-axis.

As mentioned earlier, Raman DTS yields linear temperature readings T(l), expressed as a function of distance along the fiber. Therefore, a spatial reconstruction procedure is required to convert the raw one-dimensional measurements into three-dimensional temperature fields T(x,y,z). The reconstruction methodology is presented in detail in [18], and the reconstructed temperature within the volume is illustrated in Figure 4.

The purpose of this testing facility is to conduct thermo-aeraulic experiments and to generate high-quality reference data, particularly under steady-state conditions, for the validation of thermo-aeraulic models ranging from simplified nodal approaches to full CFD simulations. To ensure the reliability of these validation datasets, it is essential to quantify and account for the measurement uncertainty associated with the distributed temperature sensing system. Consequently, each individual measurement point must be characterized not only by its reconstructed temperature value but also by its associated uncertainty, which will be incorporated into the model-validation process.

### 5.2. Strategies to Limit Sources of Uncertainty

To limit the main sources of uncertainty, several protective and operational measures were implemented throughout the measurement chain. The interrogator was installed outside the test room in a temperature-controlled environment maintained at 20 °C to minimize the influence of ambient thermal fluctuations on the instrument. Fiber attenuation was characterized, revealing low and stable attenuation for fibers embedded in the walls, whereas higher attenuation was observed for fibers installed in air. As a result, the wall-embedded fibers were characterized by a constant differential attenuation (Δα). Conversely, the air fibers were interrogated in double-ended mode, which suppresses the influence of differential attenuation along the sensing path.

To prevent accuracy degradation with distance, the total length of standard fiber embedded in the walls was limited to 1.2 km, within which the interrogator maintains stable performance for the selected integration time of 1 min. The aluminum-coated fiber installed in the reference room was restricted to 230 m and was also interrogated in double-ended mode.

Additional precautions were implemented to account for thermal exchange mechanisms affecting the fiber temperature measurement. Fibers embedded in the walls exchange heat mainly by conduction with the surrounding concrete and potentially with reinforcing elements. In contrast, fibers suspended in air exchange heat primarily by convection and, to a much smaller extent, by radiation. To detect possible thermal biases in wall-embedded fibers, localized drift checks were performed using dedicated Pt100 sensors embedded in the concrete. For fibers installed in air, a dedicated experiment was conducted to evaluate their performance in environments with significant radiative heat flux. The results demonstrated that radiation-induced measurement bias is negligible under the present experimental conditions, as presented in [5].

Based on the sources of uncertainty listed in Table 1, several contributions are considered secondary under the operating conditions of the present facility, including the effects of environmental conditions on the interrogator, interrogator attenuation, and distance-related measurement degradation along the fiber. Consequently, the measurement model for the DTS temperature can be reduced to the following additive error formulation:

(3) $$T_{measured}=T+\varepsilon_{calibr}+\varepsilon_{temporal}+\varepsilon_{spatial}+\varepsilon_{repres}+\varepsilon_{drift}+\varepsilon_{pos}$$

### 5.3. Experimental and Uncertainty Characterization

**Figure 5:**
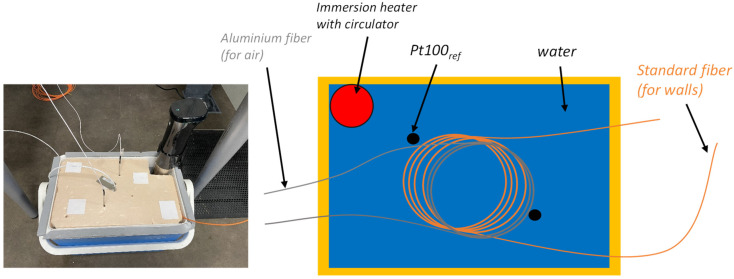
Picture and schematic of the calibration bench.

A dedicated calibration setup (Figure 5) was developed to characterize the accuracy of the complete DTS measurement chain under representative operational conditions. The interrogator–fiber system was calibrated in its final configuration and environment, using acquisition parameters identical to those employed during experiments.

Calibration was performed using a thermally insulated, well-mixed water bath covering the 20–70 °C range. Reference temperatures were provided by two class 1/10 DIN Pt100 probes located at different positions to assess bath homogeneity. For each stabilized temperature level, measurements were recorded over 30 min.

**Figure 6:**
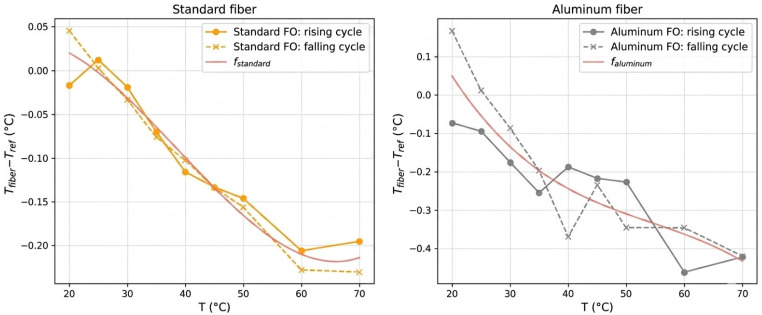
Illustration of the accuracy error for the two measurement chains and their respective correction polynomials.

The deviation between DTS measurements and the reference temperature exhibits a non-linear temperature dependence for both fibers (Figure 6), requiring the use of third-order correction polynomials. The standard fiber shows smooth bias evolution with limited hysteresis, whereas the aluminum-coated fiber presents larger dispersion, likely due to coating-induced thermo-mechanical effects. In both cases, the correction functions provide adequate compensation for the systematic bias over the calibrated range.

The calibration function was deliberately limited to a third-order polynomial in order to obtain a physically meaningful and robust estimator representative of operational conditions, and to avoid over-fitting the calibration curve to residual noise or transient effects.

The combined standard uncertainty associated with the calibrated measurement includes contributions from the reference probe calibration, bath spatial homogeneity, temporal dispersion during calibration, and the polynomial correction function. Temporal and spatial dispersion estimates are conservatively assumed to be fully correlated. The calibration uncertainty is expressed as:

(4) $$u_{calibr}=\sqrt{u_{ref}^{2}+u_{spatial}^{2}+u_{temporal}^{2}+2u_{spatial}\;u_{temporal}+{u_{function}^{calibr}}^{2}}$$

#### 5.3.1. Temporal Repeatability

The standard uncertainty associated with temporal repeatability, $u_{temporal}$, is estimated from the dispersion of temporally averaged measurements. To account for possible correlation between successive samples, the uncertainty of the mean is evaluated as:

(5) $$u_{temporal}=\sigma_{temporal}\sqrt{\frac{1+\left(n-1\right)\times r}{n}}$$

where $\sigma_{temporal}$ denotes the experimental standard deviation, $\sigma_{temporal}$ denotes the number of samples, and r is a conservative correlation coefficient.

#### 5.3.2. Spatial Dispersion

**Figure 7:**
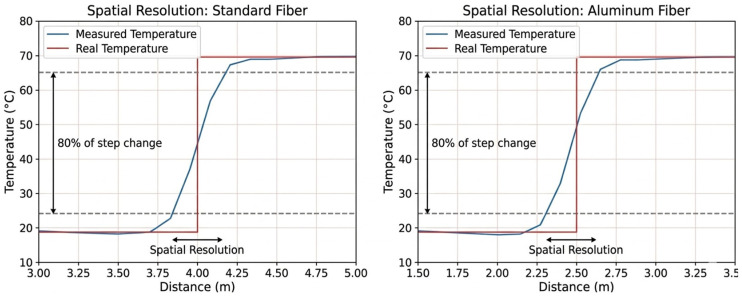
Illustration of spatial resolution for standard and aluminum optical fibers.

The spatial resolution of the interrogator was experimentally characterized for both fiber types using controlled temperature step tests. Identical responses were obtained, with full resolution of a temperature step achieved over approximately 1 m, in agreement with the manufacturer’s specifications (Figure 7).

Due to the finite spatial resolution of DTS measurements, each temperature value results from a spatially weighted contribution of neighboring fiber segments. To account for this effect in the uncertainty analysis, the interrogator response is modeled as a linear spatial filtering process, where the measured temperature profile $y\left(x\right)$ is expressed as the convolution of the true temperature field $T_{l}^{t}$ with the impulse response $h\left(x\right)$ of the measurement system:

(6) $$y\left(x\right)=h\left(x\right)*T_{l}^{t}$$

**Figure 8:**
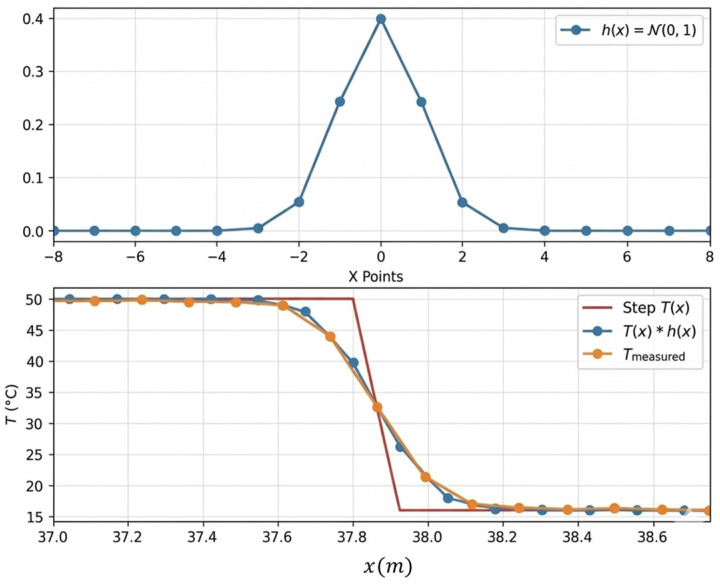
Illustration of the interrogator response (modeled and measured) to a temperature step.

Based on experimental step-response measurements, the impulse response $h\left(x\right)$ is accurately represented by a Gaussian function of zero mean, whose standard deviation is adjusted to reproduce the observed spatial spreading (i.e., full step resolution over seven sampling points). Figure 8 demonstrates the good agreement between the modeled Gaussian response and the measured DTS signal, supporting the relevance of this assumption.

This Gaussian modeling provides a physically meaningful and parsimonious description of the combined effects of optical pulse length, signal processing, and acquisition averaging inherent to the interrogator [19]. It allows the systematic estimation of the maximum spatial error induced by finite resolution without introducing additional free parameters.

To quantify the associated spatial uncertainty, two limiting temperature configurations are considered: a spatial temperature step and a spatial temperature impulse. These cases correspond to monotonic and non-monotonic local temperature gradients, respectively. The resulting spatial uncertainty is conservatively estimated using an envelope approach:

(7) $$u_{spatial}=\frac{C}{\sqrt{3}}\left(max\left[T_{i-3}:T_{i+3}\right]-min\left[T_{i-3}:T_{i+3}\right]\right)$$

where the coefficient C is set to 0.3 for monotonic gradients and 0.6 when a sign change in the local derivative is detected.

This estimator should therefore be understood as a conservative envelope derived from experimentally observed limiting cases, rather than as a universally applicable local model for arbitrary temperature profiles; its potential sensitivity to measurement noise may lead to overestimation in low-gradient conditions.

Under the experimental conditions investigated in this study, characterized by smooth spatial temperature variations both in air and in walls, the contribution of spatial resolution to the overall uncertainty remains limited.

#### 5.3.3. Measurement Chain Drift

Calibration was repeated over a three-year interval to assess long-term drift. A maximum deviation of 0.3 °C was observed at 70 °C, while drift remained below 0.1 °C over the operating temperature range of the facility (below 50 °C). Assuming a uniform distribution, the associated standard uncertainty is:

(8) $$u_{drift}=\frac{\Delta T_{drift}^{max}}{\sqrt{3}}$$

#### 5.3.4. Temperature Representativeness

The DTS does not directly measure the medium temperature but relies on thermal exchange between the fiber and its environment. Based on installation conditions and mitigation strategies adopted [18] for conduction, convection, and radiation effects, the representativeness error is estimated to remain below ±0.05 °C. The associated standard uncertainty is:

(9) $$u_{repres}=\frac{\Delta T_{repres}^{max}}{\sqrt{3}}$$

#### 5.3.5. Spatial Positioning Uncertainty

Uncertainty in fiber positioning arises from both the reconstruction method and physical placement. Given the limited spatial temperature gradients in the facility, a conservative maximum temperature deviation of 0.2 °C is assumed, leading to:

(10) $$u_{pos}=\frac{\Delta T_{pos}^{max}}{\sqrt{3}}$$

## 6. Uncertainty Propagation

Uncertainty propagation is performed using both a first-order Taylor series approximation and a Monte Carlo approach in accordance with GUM Supplement S1 [15]. Due to the similar physical origins of spatial and temporal dispersions, a full positive correlation is assumed between these two contributions.

The combined standard uncertainty associated with an instantaneous temperature measurement is given by:

(11) $$u_{T_{l}^{t}}=\sqrt{u_{calibr}^{2}+{u_{spatial}}^{2}+{u_{temporal}}^{2}+2u_{spatial}\;u_{temporal}+u_{drift}^{2}+u_{repres}^{2}+u_{position}^{2}}$$

The expanded uncertainty at a 95% confidence level is:

(12) $$U_{T_{l}^{t}}=1.96\;u_{T_{l}^{t}}$$

**Figure 9:**
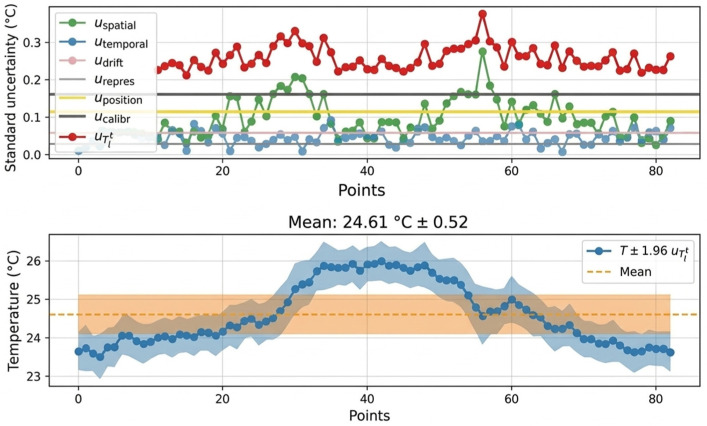
Distribution of the individual standard uncertainty components (**top**) and corresponding temperature measurement with expanded uncertainty (**bottom**) along an optical fiber segment.

Figure 9 illustrates, along a representative fiber segment embedded in the wall, the spatial distribution of the different standard uncertainty components together with the corresponding temperature profile. The calibration uncertainty appears spatially homogeneous, reflecting its global nature, whereas the spatial uncertainty exhibits local variations driven by temperature gradients along the fiber. Temporal dispersion remains nearly uniform over the considered segment and contributes less significantly under steady-state conditions. The bottom panel highlights the resulting temperature measurement with its associated expanded uncertainty envelope, showing that local variations in the combined uncertainty are primarily governed by spatial effects rather than by temporal noise or calibration drift.

**Figure 10:**
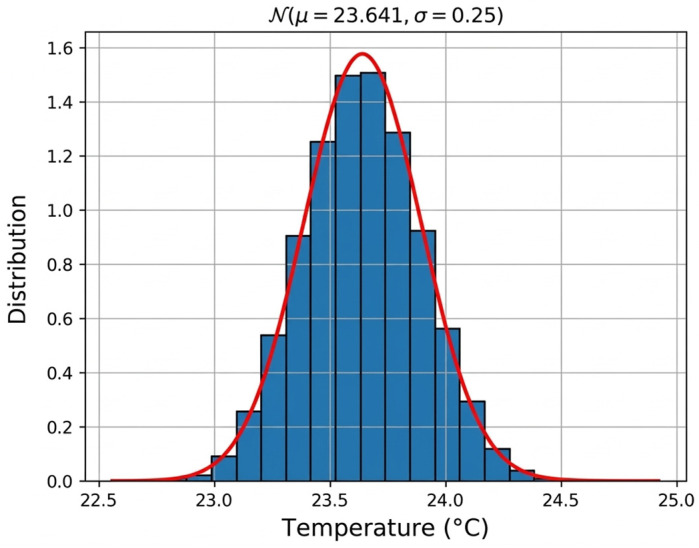
Temperature distribution of measurement points in the wall, obtained using the Monte Carlo approach.

A Monte Carlo propagation was performed on representative measurement points to validate the analytical approach. The resulting temperature distributions are Gaussian and yield expanded uncertainties consistent with those obtained from the first-order method (Figure 10). This agreement confirms the validity of the analytical uncertainty propagation model for the present application.

Table 2 summarizes the different uncertainty sources considered in the DTS temperature measurement chain together with their associated probability distributions and standard uncertainty expressions.

## 7. Uncertainty Budget and Contribution Analysis

A ranking of the contributions of the variables, noted $C_{u_{i}}$, to the variable of interest can be carried out according to the following formula:

(13) $$C_{u_{i}}=\frac{u_{i}^{2}}{u_{calibr}^{2}+u_{spatial}^{2}+u_{temporal}^{2}+u_{drift}^{2}+u_{repres}^{2}+u_{pos}^{2}}$$

**Figure 11:**
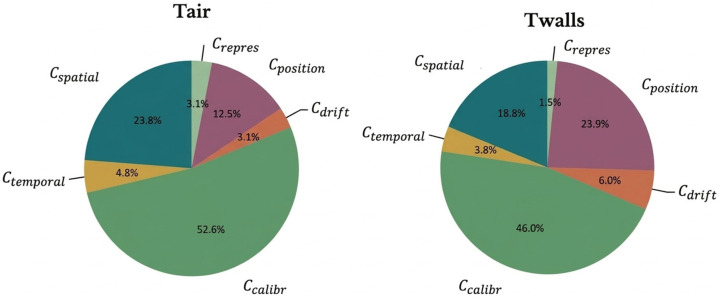
Contributions to the uncertainty.

The contribution analysis (Figure 11) highlights calibration uncertainty as the dominant term, followed by spatial dispersion and positioning uncertainty. Temporal dispersion plays a secondary role under steady-state conditions. These results confirm that, beyond calibration quality, spatial effects represent the main limiting factor in DTS-based temperature field measurements.

## 8. Conclusions

The use of Raman-based distributed temperature sensing (DTS) is becoming increasingly widespread for the characterization of temperature fields in complex environments, owing to its ability to provide spatially resolved measurements over extended volumes. However, to ensure that such measurements have genuine metrological value, a rigorous and structured evaluation of the associated uncertainties is required, a task that remains challenging due to the distributed and system-level nature of the technique.

This work proposes a complete GUM-based metrological framework for the identification, quantification, and propagation of uncertainties along the entire DTS measurement chain, from the interrogator–fiber subsystem to the reconstructed temperature fields. All significant sources of uncertainty were explicitly considered, including calibration, spatial and temporal dispersion, fiber positioning, temperature representativeness, and long-term drift. Standard uncertainty estimators were formulated for each contribution, enabling the construction of an operational and traceable uncertainty budget applicable to full-scale test facilities.

The methodology was implemented and validated on a full-scale test facility, where optical fibers are deployed to measure temperature fields both in air and within walls. Uncertainty propagation was carried out using both a first-order Taylor series approximation and Monte Carlo simulations, yielding very close results. This agreement confirms the validity of the linearized propagation model under the steady-state measurement conditions considered in this study.

The resulting uncertainty budget shows that calibration is the dominant contributor, accounting for approximately 50% of the total variance, followed by spatial dispersion and fiber positioning uncertainties, each contributing around 20%. Typical combined standard uncertainties range from 0.25 to 0.32 °C, corresponding to expanded uncertainties of approximately 0.50 to 0.63 °C at a 95% confidence level. These values demonstrate that Raman DTS can operate as a traceable temperature measurement technique when supported by a structured metrological analysis.

Particular attention was paid to spatial dispersion, whose characterization is inherently complex due to the distributed nature of DTS measurements, whereby each measurement point is influenced by neighboring fiber segments. In this work, spatial effects were modeled by representing the interrogator as a linear spatial filter, with its impulse response approximated by a Gaussian function derived from experimental step-response measurements. This approach provides a physically meaningful and robust estimator of spatial uncertainty. Although signal deconvolution using the impulse response could, in principle, allow reconstruction of the true temperature field, such methods tend to strongly amplify high-frequency noise and were therefore deemed unsuitable for the present dataset.

Beyond the establishment of a reliable uncertainty budget, this analysis highlights key improvement levers for DTS deployment in complex thermal environments, including regular system-level calibration, attenuation mitigation through double-ended configurations, and improved control of fiber routing and positioning. Future work will extend this framework to transient thermal regimes and environments with stronger radiative effects, where additional uncertainty sources and more advanced signal processing strategies may become significant.

## Figures and Tables

**Table 1:** Sources of uncertainty.

Functional Origin	Sources of Uncertainty	Symbol	Description
Interrogator	Environmental conditions	$u_{env}$	Optical and electronic components of the interrogator are sensitive to environmental conditions, particularly temperature variations.
	Internal reference measurement	$u_{offset}$	As indicated in Equation (1), the interrogator applies a temperature offset correction using an internal reference probe (typically a Pt100 probe).
	Measurement acquisition system	$u_{acq}$	The interrogator converts the optical signal into digital temperature data using optical detectors and an analog-to-digital conversion system.
Optical fiber	Differential attenuation	$u_{att}$	Differential attenuation Δα, presented in Equation (1), depends on several factors such as fiber bends, mechanical stresses, splices, temperature variations, and fiber structure (core size, coatings). These effects influence the relative intensity of the Stokes and anti-Stokes signals used to compute temperature.
	Thermal representativeness	$u_{repres}$	The fiber does not directly measure the temperature of the surrounding medium. Instead, the silica core reaches thermal equilibrium with the environment through heat transfer across the protective layers.
Interrogator-fiber system	Distance-dependent measurement quality	$u_{distance}$	The measuring chain performance may degrade with increasing distance along the fiber due to optical attenuation and signal-to-noise ratio reduction.
	Temperature-dependent variations	$u_{temperature}$	Significant changes in the medium temperature can induce biases associated with the temperature dependence of calibration parameters.
	Temporal dispersion	$u_{temporal}$	Fluctuations in the optical and electronic systems may lead to temporal variability in temperature measurements.
	Spatial dispersion	$u_{spatial}$	The spatial resolution of the DTS system and modal dispersion within the optical fiber may affect the localization of the measurement and the measured signal intensity.
	Instrument drift	$u_{drift}$	Calibration parameters such as the temperature offset $T_{offset}$ and the differential attenuation coefficient Δα may evolve over time, introducing measurement drift.
Field measurement area	Spatial positioning	$u_{pos}$	The Raman interrogator provides temperature data as a function of fiber length T(l). Converting these measurements into spatial coordinates T(x,y,z,t) requires geometric reconstruction of the fiber path, which may introduce positioning uncertainty.

**Table 2:** Standard uncertainty expressions for each source of uncertainty in the ZEPHYR testing facility.

Uncertainty Source	Distribution	Standard Uncertainty
Calibration	Normal	$u_{calibr}=\sqrt{u_{ref}^{2}+u_{spatial}^{2}+u_{temporal}^{2}+2u_{spatial}\;u_{temporal}+{u_{function}^{calibr}}^{2}}$
Spatial resolution	Uniform	$u_{spatial}=\frac{1}{\sqrt{3}}C\times \left(\max\left[T_{i-3}:T_{i+3}\right]-\min\left[T_{i-3}:T_{i+3}\right]\right)$
Temporal repeatability	Normal	$u_{temporal}=\sigma_{temporal}\sqrt{\frac{1+\left(n-1\right)\times r}{n}}$
Representativeness	Uniform	$u_{repres}=\frac{\Delta T_{repres}^{max}}{\sqrt{3}}$
Drift	Uniform	$u_{drift}=\frac{\Delta T_{drift}^{max}}{\sqrt{3}}$
Spatial positioning	Uniform	$u_{pos}=\frac{\Delta T_{pos}^{max}}{\sqrt{3}}$

## Data Availability

The data presented in this study are available from the corresponding author upon reasonable request.
